# Feeding Infants Formula With Probiotics or Milk Fat Globule Membrane: A Double-Blind, Randomized Controlled Trial

**DOI:** 10.3389/fped.2019.00347

**Published:** 2019-08-21

**Authors:** Xiaonan Li, Yongmei Peng, Zailing Li, Britt Christensen, Anne B. Heckmann, Hans Stenlund, Bo Lönnerdal, Olle Hernell

**Affiliations:** ^1^Department of Children Health Care, Children's Hospital of Nanjing Medical University, Nanjing, China; ^2^Department of Children Health Care, Children's Hospital of Fudan University, Shanghai, China; ^3^Department of Pediatrics, Peking University Third Hospital, Beijing, China; ^4^Arla Foods amba, Arla Innovation Center, Skejby, Denmark; ^5^Arla Foods Ingredients Group P/S, Viby J, Denmark; ^6^Epidemiology and Global Health, Department of Public Health and Clinical Medicine, Umeå University, Umeå, Sweden; ^7^Department of Nutrition, University of California, Davis, Davis, CA, United States; ^8^Department of Clinical Sciences, Pediatrics, Umeå University, Umeå, Sweden

**Keywords:** infant, breastfed, MFGM, F19, infection, safety, probiotics

## Abstract

**Purpose:** To evaluate effects on growth and infection rates of supplementing infant formula with the probiotic *Lactobacillus paracasei* ssp. *paracasei* strain F19 (F19) or bovine milk fat globule membrane (MFGM).

**Methods:** In a double-blind, randomized controlled trial, 600 infants were randomized to a formula supplemented with F19 or MFGM, or to standard formula (SF). A breastfed group was recruited as reference (*n* = 200).The intervention lasted from age 21 ± 7 days until 4 months, and infants were followed until age one year.

**Results:** Both experimental formulas were well tolerated and resulted in high compliance. The few reported adverse events were not likely related to formula, with the highest rates in the SF group, significantly higher than for the F19-supplemented infants (*p* = 0.046). Weight or length gain did not differ during or after the intervention among the formula-fed groups, with satisfactory growth. During the intervention, overall, the experimental formula groups did not have more episodes of diarrhea, fever, or days with fever than the breastfed infants. However, compared to the breastfed infants, the SF group had more fever episodes (*p* = 0.021) and days with fever (*p* = 0.036), but not diarrhea. Compared with the breastfed group, the F19-supplemented infants but not the other two formula groups had more visits/unscheduled hospitalizations (*p* = 0.015) and borderline more episodes of upper respiratory tract infections (*p* = 0.048).

**Conclusions:** Both the MFGM- and F19-supplemented formulas were safe and well-tolerated, leading to few adverse effects, similar to the breastfed group and unlike the SF group. During the intervention, the MFGM-supplemented infants did not differ from the breastfed infants in any primary outcome.

## Introduction

Breastfeeding is considered the “gold standard” for infant nutrition because human milk offers an adequate supply of nutrients and biologically active components with benefits for growth, development, and protection against infections (1). Infants fed standard formula (SF) are at higher risk of otitis media (2) and gastrointestinal and respiratory infections (3, 4). For this reason, a goal of infant formula development is to emulate the composition and functionality of breast milk to close this gap in health outcomes (5). Anti-infectious factors in human milk include immunoglobulins, anti-bacterial and anti-viral proteins, leukocytes, and oligosaccharides, which collectively are considered to reduce the risk of gastrointestinal and other infections in breastfed infants. Studies also strongly suggest that the gut microbiota is associated with positive health outcomes (6, 7). Diet is among the main drivers of the composition and function of the gut microbiota (8). In breastfed infants, bifidobacteria and lactobacilli dominate this microbiota, whereas formula-fed infants have a more diverse colonization, including *Bacteroidetes*, bifidobacteria, staphylococci, *Escherichia coli*, and *Clostridia* (9–12). Several meta-analyses have reported that supplementation with a probiotic may be beneficial in preventing and treating upper respiratory tract infections (13), infectious diarrhea, and antibiotic-induced diarrhea (14), as well as allergic disease, e.g., eczema in children (15). Some studies, however, have found no effect of probiotics (16–18). It seems reasonable to develop infant formulas that support establishment of a microbiota resembling that of breastfed infants through the addition of bioactive components or probiotics. A previous study indicated that supplementing with the *Lactobacillus paracasei* ssp. *paracasei* strain F19 (F19) during weaning could be an effective tool in prevention of early manifestations of allergy, such as eczema, in infants ages 4–13 months (19). Results of another study suggested a reduced risk of lower respiratory tract infections when this probiotic was combined with prebiotics (20). Collectively, these studies support that F19 is safe, even from the first months of life.

The milk fat globule membrane (MFGM) envelops the triglyceride-rich core of the milk fat globule when secreted from epithelial cells of the lactating mammary gland. This membrane contains numerous biologically active components (21, 22), many with antimicrobial effects, e.g., gangliosides (23), oligosaccharides (24), and the glycoproteins butyrophilin, lactadherin, and mucin (25, 26). By tradition, infant formulas have been produced from skim milk powder and whey protein concentrate, and the milk fat has been discarded. The fat is typically replaced by a blend of vegetable oils. For this reason, compared to breast milk, infant formulas contain much less of the biologically important MFGM proteins and lipids. Results of a growing number of clinical trials of MFGM supplementation for infants or children support positive effects on both neurodevelopment (27, 28) and defense against infections (29, 30). Bovine milk fractions enriched in MFGM are now commercially available, and infant formulas with MFGM have been launched in several countries.

The aim of the present study was to evaluate the effects of feeding infants a SF supplemented with either F19 or MFGM compared to feeding them unsupplemented SF, and using a breastfed group as reference with regard to infant growth and health. The primary hypothesis was that consumption of formula containing either F19 or MFGM would reduce the incidence of infections. Furthermore, we hypothesized that feeding infant formula with F19 or MFGM from the first months of life would be safe and tolerable.

## Methods

The study was conducted at several centers in China in Nanjing (Children's Hospital of Nanjing Medical University, Nanjing Maternity and Child Health Care Hospital, the Second Affiliated Hospital of Nanjing Medical University, Nanjing Secondary Hospital, and Huaian Maternity and Child Health Hospital), Shanghai (Children's Hospital of Fudan University, Clinical Center for Public Health of Fudan University), and Beijing (Peking University Third Hospital, Beijing Ditan Hospital Capital Medical University, and The First Hospital of Jilin University). It was approved by the institutional review board at the University of California, Davis, as well as the regional ethical review boards in Nanjing, Shanghai, and Beijing, China, and conducted according to the principles in the Declaration of Helsinki. Complete oral and written information about the study was given to the parents/caregivers, and written consent was obtained from the parents or caregivers of all infants before inclusion. The clinical trial was registered at ClinicalTrials.gov (NCT01755481).

### Inclusion Criteria and Background Information

The study was a randomized, double-blind, controlled trial comparing three different infant formulas, with breastfed infants as the reference group. Statistical power calculations revealed that a sample size of 540 infants (180 in each group) was needed to detect a difference of 20% in incidence of infectious episodes, the primary outcome, with 80% power (5% significance). Anticipating a drop-out rate of 15–20%, our aim was to include 800 infants, 200 in each formula group and 200 breastfed infants. Infants were recruited consecutively from December 2013 to August 2016. Inclusion criteria for all infants were gestational age of 37–42 weeks at birth, birth weight >2,500 g and <4,000 g, absence of chronic illness, and a parent or legal representative who could speak and understand Chinese. Exclusion criteria for all infants were malformations, handicaps, or congenital diseases that could affect normal feeding or growth, treatment with antibiotics (including perinatal treatment of the mother), and having been fed infant formula with pre- and/or probiotics. Inclusion criteria for the formula-fed group were healthy infants of mothers who could not or voluntarily completely refrained from breastfeeding at inclusion (infant age 21 ± 7 days). The exclusion criterion for the formula-fed groups was any breastfeeding at the age of 28 days. Inclusion criterion for the breastfed group was having been exclusively breastfed from birth and mothers intending to breastfeed >80% to age at least 4 months. Exclusion criteria for the breastfed group were infants fed >20% infant formula of their calculated total intake at 28 days of age. Background information was collected at the time of recruitment. Information on birth weight, feeding pattern, and parental education was recorded for all excluded and drop-out infants.

### Composition of Infant Formulas

Formulas were manufactured from bovine milk powder by Arla Foods amba, Denmark. The probiotic bacterium *L. paracasei*, ssp*. paracasei* strain F19 was from Chr. Hansen, Denmark, and Lacprodan^®^ MFGM-10 from Arla Foods Ingredients group P/S, Denmark. The composition of each of the three formulas is shown in Table 1. The final study formulas were produced in Hohhot, China, in accordance with Chinese regulations under strict hygienic conditions, adhering to all prerequisites for human consumption.

**Table 1:** Composition of infant formulas used in the study.

	**SF^a^**	**MFGM**	**F19**
**Energy** (kcal/100 mL)	66	67	66
**Protein** (g/100 mL)	1.6	1.5	1.6
Casein (g/100 mL)	0.60	0.59	0.60
Whey (g/100 mL)	0.99	0.95	0.97
**Carbohydrate** (g/100 mL)	7.0	7.3	7.0
**Fat** (g/100 mL)	3.5	3.6	3.5
Linoleic acid (g/100 mL)	0.7	0.7	0.7
α-Linolenic acid (mg/100 mL)	64	65	64
DHA^b^ (% of total fatty acids)	0.33	0.31	0.29
ARA (% of total fatty acids)	0.45	0.43	0.39
**Minerals**			
Sodium (mg/100 mL)	20	20	20
Potassium (mg/100 mL)	67	62	67
Copper (μg/100 mL)	61	59	61
Magnesium (mg/100 mL)	8.1	8.0	8.1
Iron (mg/100 mL)	0.81	0.81	0.81
Zinc (mg/100 mL)	0.6	0.6	0.6
Manganese (μg/100 mL)	8.5	8.1	8.5
Calcium (mg/100 mL)	50	49	50
Phosphorus (mg/100 mL)	36	34	36
Iodine (μg/100 mL)	11	12	11
Chloride (mg/100 mL)	47	48	47
Selenium (μg/100 mL)	2.5	2.3	2.5
**Vitamins**			
Vitamin C (mg/100 mL)	9.1	9.3	9.1
Vitamin A (μgRE/100 mL)	85	81	85
Vitamin E (mg α-TE/100 mL)	1.2	1.2	1.2
Vitamin D (μg/100 mL)	1.0	1.1	1.0
Vitamin K1 (μg/100 mL)	5.4	5.2	5.4
Vitamin B1 (μg/100 mL)	86.1	88.0	86.1
Vitamin B2 (μg/100 mL)	211	159	211
Vitamin B6 (μg/100 mL)	80.3	72.4	80.3
Vitamin B12 (μg/100 mL)	0.4	0.3	0.4
Niacin (μg/100 mL)	742	761	742
Folic acid (μg/100 mL)	17.0	15.7	17.0
Pantothenic acid (μg/100 mL)	644	575	644
Biotin (μg/100 mL)	2.8	2.4	2.8
**Optional ingredients**			
Choline (mg/100 mL)	10.7	8.9	10.7
Inositol (mg/100 mL)	4.6	4.8	4.6
Lutein (μg/100 mL)	9.6	8.8	9.6
Nucleotide^c^ (mg/100 mL)	2.9	3.0	2.9
Taurine (mg/100 mL)	6.2	5.8	6.2
L-carnitine (mg/100 mL)	1.9	1.7	1.9

a *SF, standard formula; MFGM, formula supplemented with Lacprodan^®^ MFGM-10; F19, formula supplemented with L. paracasei ssp. paracasei strain F19*.

b *DHA, docosahexaenoic acid; ARA, arachidonic acid*.

c *A mixture of disodium salts of 5'-AMP, 5'-CMP, 5'-GMP, 5'-UMP, and 5'-IMP*.

### Randomization and Intervention

The intervention was blinded both to parents and staff until analyses were completed. Infants were randomized to one of the three infant formulas: SF; the same formula supplemented with F19 at a dose of 1^*^10^8^ cfu/L; or Lacprodan^®^ MFGM-10 (3.88 g Lacprodan^®^ MFGM-10/100 g powder, or 5 g/L prepared formula) from inclusion at 21 ± 7 days to the end of the fourth month. For randomization, a computerized randomization tool in blocks of 24 was used, stratifying for sex (12 boys and 12 girls) and type of formula coded by color (eight of each color). The block size for the breastfed group was eight (4 boys and 4 girls) in each group. Powdered formula was distributed to families together with preparation instructions in identical boxes marked with a color coded number, prepared at the manufacturing site before being sent to the study site. Prior to the start of intervention, infants were fed SF if formula feeding had been started.

From the beginning of the fifth month to the end of the sixth month of age, all infants in the formula groups received SF. If breast milk supply was insufficient, breastfed infants were fed SF, but not exceeding 20% of their calculated total intake based on the 3-day formula intake record (see below). Complementary foods were not allowed during the intervention but were introduced no later than 26 weeks of age, according to current recommendations. Vitamin D supplements were given according to current recommendations.

### Assessment of Growth

Visits were made at baseline (inclusion) and at 1, 2, 3, 4, 5, 6, 9, and 12 months of age. At each visit, weight, length, and head circumference were measured. Weight was assessed to the nearest 10 g. The same electronic weighing scales (Seca 757; Seca, Germany) were used for all infants at all visits at each center and calibrated at the first visit and every visit thereafter until the end of the study. Recumbent length was measured to nearest 1 mm using a standardized length board (Seca 416; Seca, Germany). Head circumference was measured to the nearest 1 mm using a standard non-elastic plastic-coated measuring tape (Seca 212, Seca, Germany). Anthropometric data are presented as z-scores calculated from the World Health Organization reference growth standards for breastfed infants (31).

### Formula Intake

Parents of formula-fed infants were asked to complete a 3-day formula intake diary every month from inclusion until the end of the fifth month of age. Parents or caregivers of breastfed infants were asked to note all formula fed to the infant to allow the study staff to check adherence with breast milk consumption.

### Assessment of Episodes of Infections and Health

From inclusion until 12 months of age, the incidence and duration of infectious episodes (acute diarrhea, upper and lower acute respiratory tract infections, fever) were diagnosed and recorded by the study physician based on the following definitions: Acute diarrhea was defined as three or more watery stools within a 24-h period or loose-to-watery bowel movements that exceeded the infant's usual daily stool frequency by two or more stools. Acute respiratory infections were defined as presence of two or more of the following symptoms as reported by the parent/caretaker: nasal discharge (clear, cloudy, yellow, or green), cough, fever, rapid, labored and/or noisy breathing, wheezing, chest in-drawing, flaring of nostrils, ear pain and/or discharge, and cyanosis. Respiratory symptoms that occurred within 2 weeks of the beginning of the illness were defined as part of the same episode. Symptoms presented more than 2 weeks after the start of an incident were considered as a new episode. Parent-reported number of episodes and days with fever (>38°C), vomiting, use of antibiotics, unscheduled doctor's visits, and hospitalization (incidence, duration, diagnosis, and treatment) were registered based on reviews performed every second week by the study physician. The length of the period of antibiotic use was recorded. Stool consistency was registered as watery diarrhea, loose, soft formed, or hard in the monthly 3-day dietary and health record. Parents/caretakers whose infants dropped out were asked to remain in the study for follow-up on an intention-to-treat basis.

### Definition of Adverse Event and Serious Adverse Event

An adverse event (AE) was defined as any untoward occurrence in an infant administered a test product and that did not necessarily have to have a causal relationship with the product. AEs were illnesses, signs, or symptoms (including an abnormal laboratory finding) occurring or worsening during the course of the study. A serious adverse event (SAE) was a fatal or life-threatening event causing permanent harm or requiring/extending inpatient treatment at a hospital, or that the physician considered medically relevant.

All AEs were documented on the case report form. In the case of a SAE persisting beyond the trial termination, a follow-up visit was required. Furthermore, study physicians analyzed each report for a potential cause–effect relationship between the study products and the AE. Cow's milk protein allergy was diagnosed by a physician as follows: elimination of cow's milk protein/formula with disappearance of symptoms and reappearance of the same symptoms on reintroduction of milk protein/formula. When diagnosed, infants were recommended a protein hydrolysate formula and were considered study drop-outs.

### Blood Sample Collection and Storage

All infants had a venous blood sample of 0.5–2 mL collected by the study staff on two occasions, one at the end of the intervention when the infants were age 4 months. After centrifugation at 1,500 rpm for 10 min, serum was collected and immediately frozen and stored at −80°C until shipped on dry ice to the sites of analyses.

### Stool Sample Collection and Storage

Parents/legal representatives were asked to collect stool samples at different time points, including at the end of the intervention period. Before collection of the first stool sample, a reusable isolated bag for transportation of the stool samples, a reusable freezing body, two containers for the stool samples, a plastic bag for the filled containers, gloves, and instructions for collection, storage, and transportation of the stool samples were provided to the parent or the infant's legal representative. Stool samples were collected in the containers, put in the plastic bag, and stored in a freezer (−20°C) until the day of the visit. The frozen stool samples were transported to the study site, where they were stored at −20°C until shipped on dry ice for analysis (TNO, The Hague, Netherlands).

### Serum Ferritin

Serum ferritin was analyzed in infants at age 4 months, with an enzyme-linked immunosorbent assay kit (RayBiotech, Norcross, GA, USA). This kit uses a biotinylated antibody specific for human ferritin and horseradish peroxidase–conjugated streptavidin. Samples were run in duplicate, and values are presented as means.

### Fecal DNA Extraction and qPCR

DNA from fecal samples collected at the end of the intervention was isolated as previously described (32) with some minor modifications. The samples were initially mixed with 250 μL lysis buffer (Agowa, Berlin, Germany), 250 μL zirconium beads (0.1 mm), and 200 μL phenol, before being introduced to a BeadBeater (BioSpec Products, Bartlesville, OK, USA) for two × 2 min. Quantitative PCR detection was performed with the primers and according to conditions described previously (33). To evaluate if samples contained F19, data were plotted from low to high Ct, resulting in an S-shaped curve, with true positives in the lower and true negatives in the higher end, as previously described (33).

### Statistics

Comparison of means among the MFGM, and F19 groups and the SF group were done pair-wise with independent samples *t*-tests. In the case of skewed distributions, comparisons were performed with Mann–Whitney *U*-test. Categorical variables were compared pair-wise using the Chi square test or Fisher's exact test. Comparisons of treatment groups with the breastfed group were also done pair-wise. Analysis of variance was used for analyzing ferritin concentrations among groups.

Only adjusted *p*-values are presented in the text. Adjustment was based on the Bonferroni method. Per-protocol analyses were based on the 674 children who completed the study. There were no significant differences among the three sites with respect to birth weight, sex distribution, or education level, or with respect to major outcomes. All calculations were done using SPSS v 23 (IBM SPSS Statistics, Armonk, NY). Significance level was set to 5%.

## Results

**Figure 1:**
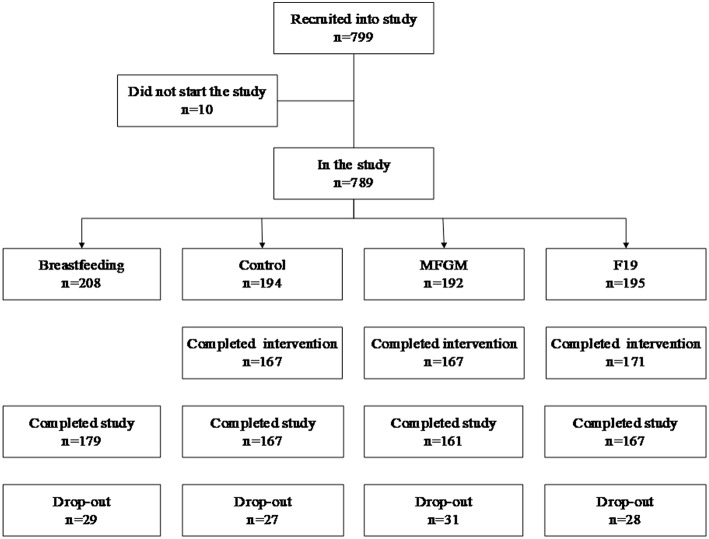
Drop-out rates and adherence to the intervention in the formula-fed groups (SF, standard formula; MFGM, formula supplemented with milk fat globule membrane; F19, formula supplemented with *L. paracasei* ssp*. paracasei* strain F19) and the BF (breastfed) group. Ten children did not show up at any of the examinations and were excluded. During the study, 115 children left the study at various times, giving a total drop-out rate of 14.6% (BF, 14.1; SF, 14.1; MFGM, 17.9, and F19, 15.1%) with no significant difference among groups. The most common reason for drop-out (78%) was parent/caregiver decision to do so without explanation.

In total, 799 children were recruited and randomized to formula-fed groups or recruited to the breastfed reference group. Ten infants did not attend any of the visits. Thus, 789 children remained for intention-to-treat analyses. In these analyses, missing continuous values were replaced according to “carry forward last value.” Missing categorical values were replaced with zero (Figure 1).

### Adherence

During the intervention, no formula consumption was reported for 22 formula-fed children, 42 children had consumption reported for 1, 2, or 3 months, and 505 children had consumption reported for the whole intervention. The proportion of children adherent for the whole intervention period did not differ significantly among the formula-fed groups. Average formula consumption during the intervention for the SF, MFGM, and F19 groups was 876, 866, and 833 mL/day, respectively. The F19 group consumed a lower average volume than the SF group (*p* = 0.020), but there was no difference between the SF and MFGM groups. Analysis of F19 in the stool in a randomized subsample of 100 from each of the formula-fed groups at age 4 months showed that 92% of the infants in the F19 group carried F19 compared to none in the other formula groups, confirming high adherence (data not shown).

### Demographic Characteristics

Basic characteristics for the groups are shown in Table 2. There was no significant difference in birth weight, sex distribution, gestational age, type of delivery, pregnancy complications, parental education level, or proportion of no siblings among the formula-fed groups. However, birth weight (*p* = 0.002), parental education level (*p* < 0.013), and proportion of no siblings (*p* = 0.001) were lower for the formula-fed groups combined than for the breastfed group.

**Table 2:** Demographic characteristics of the formula-fed and breastfed groups.

**Variables**	**BF**	**SF**	**MFGM**	**F19**
	**(*n* = 208)**	**(*n* = 194)**	**(*n* = 192)**	**(*n* = 195)**
Birth weight (g), mean (SD)	3381 (314)	3298 (374)	3279 (399)	3281 (412)
Sex, girls (%)	52.9	49.5	51.6	49.7
Gestational age (weeks)	39.0	38.7	38.8	38.8
Siblings (% with no siblings)	75.5	61.9	64.1	61.5
Delivery (% cesarean)	45.9	58.8	62.8	57.5
Pregnancy complications (%)	7.7	9.8	9.4	7.2
Mother's age (years), mean (SD)	29.4 (3.5)	29.6 (4.6)	29.2 (4.3)	29.5 (4.5)
Father's age (years), mean (SD)	31.3 (4.4)	31.4 (5.2)	31.1 (4.9)	31.6 (6.1)
Mother's education (%)	4.3/25.0/70.7	9.8/47.9/41.2	9.9/44.3/44.8	9.2/40.5/46.7
Father's education (%)	3.8/21.2/74.5	9.3/47.4/42.3	7.3/41.7/49.0	9.7/43.6/44.6

*BF, breastfed reference group; SF, standard formula; MFGM, formula supplemented with milk fat globule membrane; F19, formula supplemented with L. paracasei ssp. paracasei strain F19; SD, standard deviation*.

*Education (%) refers to low/middle/high education level, i.e., ≤ 12 y/13–15y/ ≥ 16 y of school and university education*.

### Anthropometrics

**Figure 2:**
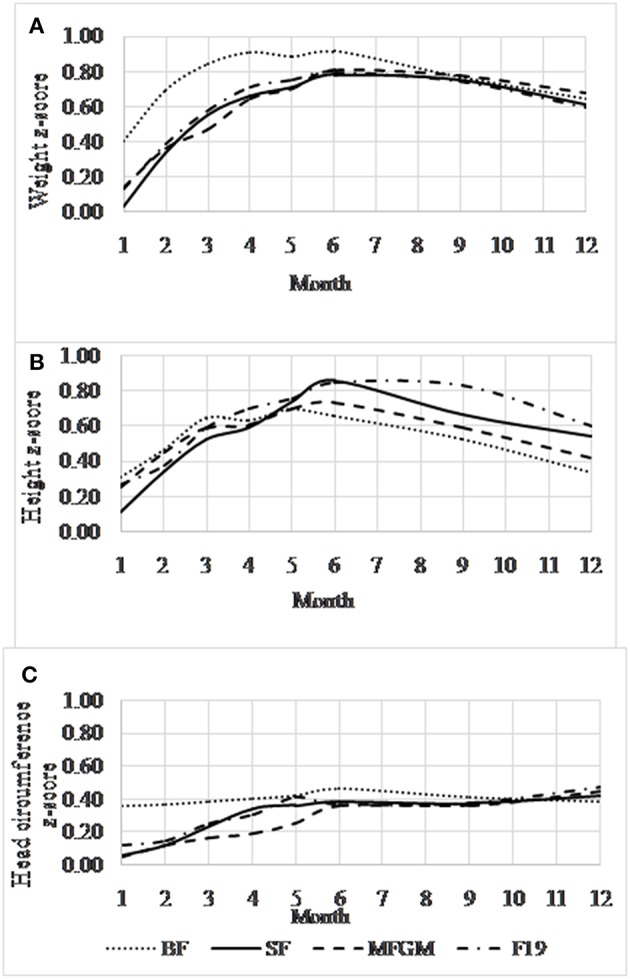
Mean (95% confidence interval) age-adjusted anthropometric z-score data (y-axis) for the formula-fed and breastfed groups. Weight for age **(A)**, length for age **(B)**, and head circumference for age **(C)** using the World Health Organization reference population (31). BF, breastfed; SF, standard formula; MFGM, formula supplemented with milk fat globule membrane; F19, formula supplemented with *L. paracasei* ssp. *paracasei* strain (F19). There were no significant differences among the formula-fed groups in mean z-scores at any time point or for any of the growth variables.

Weight z-scores did not differ significantly among the formula-fed groups at any time point. Mean weight for the breastfed group was significantly higher than for the F19 group until age 2 months (*p* = 0.015 and 0.028 at 1 and 2 months, respectively) and for the SF and MFGM groups until age 4 months (all *p* ≤ 0.041). After these ages, the groups showed no significant differences (Figure 2A and Table 3). During the intervention, weight gain (g/day) did not differ among the formula-fed groups or between the formula-fed groups overall and the breastfed group. However, at 5–12 months, weight gain in the MFGM group was slightly (1.1 g/day) but significantly higher compared to the breastfed group (*p* = 0.012) (Table 4).

**Table 3:** Weight, height and head circumference from 1 to 12 months of age.

	**BF**	**SF**	**MFGM**	**F19**	**SF vs. MFGM**	**SF vs. F19**
	**(*****n*** **=** **208)**	**(*****n*** **=** **194)**	**(*****n*** **=** **192)**	**(*****n*** **=** **195)**	***p*****-values**	***p*****-values**
**WEIGHT (KG)**						
1 month	4.6	4.4	4.4	4.4	0.612	0.532
2 months	5.8	5.6	5.6	5.7	0.999	0.746
3 months	6.8	6.6	6.5	6.6	0.836	0.999
4 months	7.5	7.3	7.2	7.3	0.999	0.999
5 months	8.0	7.8	7.8	7.8	0.999	0.999
6 months	8.4	8.4	8.4	8.4	0.999	0.999
9 months	9.4	9.4	9.4	9.4	0.999	0.999
12 months	10.2	10.1	10.2	10.1	0.900	0.999
**HEIGHT (CM)**						
1 month	54.8	54.4	54.7	54.7	0.432	0.310
2 months	58.7	58.5	58.6	58.5	0.828	0.999
3 months	61.9	61.7	61.8	61.8	0.999	0.999
4 months	64.3	64.3	64.2	64.5	0.999	0.780
5 months	66.4	66.6	66.4	66.6	0.999	0.999
6 months	68.1	68.6	68.3	68.5	0.390	0.999
9 months	72.2	72.6	72.4	73.0	0.848	0.240
12 months	75.7	76.2	75.9	76.4	0.472	0.999
**HEAD CIRCUMFERENCE (CM)**						
1 month	37.3	37.0	37.0	37.0	0.999	0.999
2 months	39.1	38.8	38.8	38.9	0.999	0.999
3 months	40.4	40.3	40.2	40.3	0.802	0.999
4 months	41.6	41.5	41.3	41.4	0.292	0.590
5 months	42.5	42.5	42.4	42.5	0.999	0.999
6 months	43.3	43.2	43.2	43.2	0.956	0.999
9 months	44.9	44.9	44.8	44.9	0.999	0.999
12 months	46.0	46.1	46.1	46.1	0.999	0.999

*BF, breastfed reference group; SF, standard formula; MFGM, formula supplemented with milk fat globule membrane; F19, formula supplemented with Lactobacillus paracasei ssp. paracasei strain F19*.

**Table 4:** Mean weight/length/head circumference gain during 0–4 months and 5–12 months.

	**BF**	**SF**	**MFGM**	**F19**	**SF vs. MFGM**	**SF vs. F19**
	**(*****n*** **=** **208)**	**(*****n*** **=** **194)**	**(*****n*** **=** **192)**	**(*****n*** **=** **195)**	***p*****-values**	***p*****-values**
**0–4 MONTHS**						
Weight gain (g/day)	31.5	31.7	30.9	31.7	0.508	0.999
Length gain (cm/day)	0.104	0.107	0.104	0.106	0.276	0.999
Head circumference (cm/day)	0.047	0.050	0.048	0.047	0.698	0.912
**5–12 MONTHS**						
Weight gain (g/day)	10.3	10.8	11.4	10.9	0.224	0.999
Length gain (cm/day)	0.044	0.045	0.044	0.046	0.656	0.999
Head circumference (cm/day)	0.017	0.017	0.017	0.017	0.112	0.999

*BF, breastfed reference group; SF, standard formula; MFGM, formula supplemented with milk fat globule membrane; F19, formula supplemented with Lactobacillus paracasei ssp. paracasei strain F19*.

Z-scores for body length did not differ significantly among the formula-fed groups at any time point (Figure 2B and Table 3). The SF and MFGM groups did not differ significantly from the breastfed group at any time point, but the F19 infants had significantly greater length at ages 9 (*p* = 0.009) and 12 months (*p* = 0.048). Gain in body length (cm/day) did not differ among any of the groups during or after the intervention (Table 4).

Head circumference z-scores did not differ significantly among the formula-fed groups at any time between 1 and 12 months (Figure 2C and Table 3). During the intervention, the breastfed group had larger head circumference than all of the formula-fed groups at 1 and 2 months (MFGM, *p* = 0.012 and *p* = 0.048, respectively; F19, *p* = 0.051 and 0.042, respectively; SF, *p* = 0.003 and 0.024, respectively). After the intervention, the groups showed no differences in head circumference or in gains in head circumferences (Table 4).

### Primary Outcomes

During the intervention, both the MFGM and the F19 groups had numerically fewer episodes of fever (>38°C) and days with fever than the SF group, although the differences did not reach statistical significance. However, compared to the breastfed group, the SF infants had significantly more fever episodes (*p* = 0.021) and days with fever (*p* = 0.036), but not episodes of diarrhea. In contrast, neither the MFGM nor the F19 groups had significantly more episodes of fever or number of days with fever than the breastfed group (Table 5).

**Table 5:** Primary outcomes during 0–4 months and 5–12 months.

	**BF**	**SF**	**MFGM**	**F19**	**SF vs. MFGM**	**SF vs. F19**
	**(*****n*** **=** **208)**	**(*****n*** **=** **194)**	**(*****n*** **=** **192)**	**(*****n*** **=** **195)**	***p*****-values**	***p*****-values**
**0–4 MONTHS**						
Diarrhea (episodes)	14	9	7	15	0.999	0.532
Fever > 38°C (episodes)	6	21	11	10	0.242	0.214
Days with fever	8	31	18	11	0.230	0.122
Other infections (episodes)						
URI	18	27	28	33	0.999	0.966
LRI	0	5	2	8	0.898	0.999
**5–12 MONTHS**						
Diarrhea (episodes)	25	34	45	36	0.422	0.999
Fever >38°C (episodes)	77	85	94	79	0.908	0.999
Days with fever	179	197	224	185	0.999	0.999
Other infections (episodes)						
URI	82	82	104	101	0.050	0.136
LRI	3	5	3	8	0.999	0.999

*BF, breastfed reference group; SF, standard formula; MFGM, formula supplemented with milk fat globule membrane; F19, formula supplemented with Lactobacillus paracasei ssp. paracasei strain F19; URI, upper respiratory tract infection; LRI, lower respiratory tract infection*.

During the intervention, the number of episodes of upper respiratory tract infections did not differ among the formula-fed groups or between these groups overall and breastfed infants; however, the F19 group had more episodes than the breastfed group (*p* = 0.048). The F19 infants also had more episodes of lower respiratory tract infections than the breastfed group (*p* = 0.009).

During the post-intervention period (5–12 months), the formula-fed groups did not differ significantly for any of the primary outcomes except for marginally more episodes of upper respiratory tract infections for the MFGM infants compared to the SF group (*p* = 0.050). Compared to the breastfed infants, the MFGM group had more episodes of diarrhea (*p* = 0.021) (Table 5). Per-protocol analyses of the primary outcomes did not differ from the intention-to-treat analyses (data not shown).

### Secondary Outcomes

During the intervention, the formula-fed groups did not differ with respect to skin affections, use of antibiotics, vomiting, or unscheduled visits/hospitalizations (Table 6). However, compared to the breastfed group, the F19 infants used significantly more antibiotics and had more unscheduled visits and/or hospitalization episodes (*p* = 0.045 and 0.015, respectively). After the intervention, only the F19 group used more antibiotics compared to the breastfed infants (*p* = 0.003), with no difference among the formula-fed groups. None of the formula-fed infants had more unscheduled visits/hospitalizations than the breastfed group after the intervention.

**Table 6:** Secondary outcomes during 0–4 months and 5–12 months.

	**BF**	**SF**	**MFGM**	**F19**	**SF vs. MFGM**	**SF vs. F19**
	**(*****n*** **=** **208)**	**(*****n*** **=** **194)**	**(*****n*** **=** **192)**	**(*****n*** **=** **195)**	***p*****-values**	***p*****-values**
**0–4 MONTHS**						
Skin effects	17	20	16	16	0.999	0.980
Use of antibiotics	7	21	9	19	0.128	0.999
Vomiting	0	0	1	0	–	–
Unscheduled visits and/or hospitalization	9	23	15	25	0.986	0.798
**5–12 MONTHS**						
Skin infections	4	0	2	0	0.480	–
Use of antibiotics	33	50	53	65	0.999	0.506
Vomiting	0	1	0	0	–	–
Unscheduled visits and/or hospitalization	45	54	55	70	0.720	0.708

*BF, breastfed reference group; SF, standard formula; MFGM, formula supplemented with milk fat globule membrane; F19, formula supplemented with Lactobacillus paracasei ssp. paracasei strain F19*.

### Adverse Events

The number of AEs was low, with no differences within any of the reported categories among the formula-fed groups or between breastfed and formula-fed infants during the intervention or the post-intervention period (Table 7). However, during the intervention, the total number of AEs was highest in the SF group and significantly higher than in the F19 group (*p* = 0.046), but with no significant difference after the intervention.

**Table 7:** Adverse events during 0–4 months and 5–12 months.

	**BF**	**SF**	**MFGM**	**F19**	**SF vs. MFGM**	**SF vs. F19**
	**(*****n*** **=** **208)**	**(*****n*** **=** **194)**	**(*****n*** **=** **192)**	**(*****n*** **=** **195)**	***p*****-values**	***p*****-values**
**0–4 MONTHS**						
Oral infections	1	2	1	1		
Gastrointestinal infections^a^	1	4	3	0		
Other viral infections	1	0	0	0		
Other bacterial infections	0	0	1	0		
Hematochezia	0	2	0	0		
Constipation	1	2	1	0		
Other non-infectious diseases	0	1	0	0		
Skin effects	1	4	1	4		
Total	5	15	7	5	0.140	0.046
**5–12 MONTHS**						
Oral infections	5	2	0	2		
Gastrointestinal infection^a^	0	1	0	0		
Other viral infections	5	4	8	3		
Other bacterial infections	2	0	0	1		
Hematochezia	0	0	0	0		
Constipation	0	0	0	0		
Other non-infectious diseases	0	0	1	2		
Skin effects	1	0	0	0		
Total	13	7	9	8	0.999	0.999

*BF, breastfed reference group; SF, standard formula; MFGM, formula supplemented with milk fat globule membrane; F19, formula supplemented with Lactobacillus paracasei ssp. paracasei strain F19*.

a *Not diarrhea*.

In total, 20 infants had SAEs, and one infant had two events. The SAEs were distributed among the groups as follows: breastfed, four; SF, four; MFGM, seven; and F19, six. The most common SAE was lower respiratory tract infection, with 16 episodes. During the intervention period, there were six SAEs, three among breastfed infants, one in the MFGM group, and two in the F19 group.

Of all the reported AEs and SAEs, 12 were considered probably related to the formula and one definitely related by the responsible pediatrician. Eleven of the infants developed skin affections and one infant constipation. Four of the infants had no treatment for their skin affections, five had local treatment with lubricant and/or steroids, and one infant also local antibiotics. The formula was switched to another formula by the parents of one infant (constipation), and 3 days later this infant dropped out from the study. For another infant, the formula was switched to a partially hydrolyzed formula. This infant had a skin infection and was treated with local steroids and antibiotics. Whether the switch of formula had an effect is unknown. For a third infant, formula was also withdrawn, and the mother went back to exclusive breastfeeding. The only infant for whom the skin affection was classified as definitely related to the formula stopped eating the formula, but the diagnosis was never proven by challenging the infant with the formula. This infant belonged to the MFGM group. The 12 infants experiencing these events were distributed across all groups: SF, four; F19, four; MFGM, two; and breastfed, two. Thus, the AEs possibly related to the formula were few and not proven in any of the infants, and there was no significant difference among the groups.

### S-Ferritin

**Figure 3:**
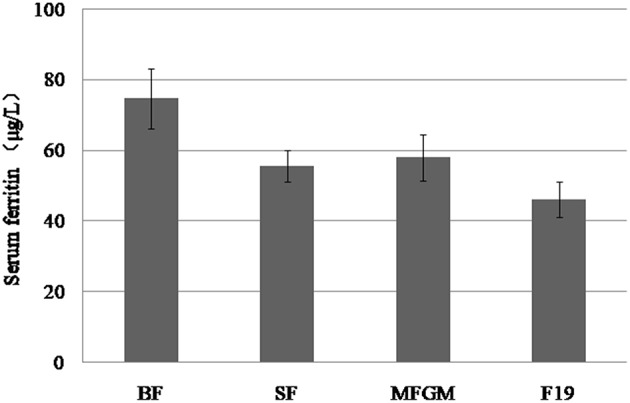
Serum ferritin levels in the breastfed group and in the formula-fed groups. S-ferritin concentration was analyzed in a random sample consisting of 50 infants from each of the four groups. There was no difference in s-ferritin concentration among the formula-fed groups, but the F19 group was significantly lower than the breastfed group (*p* = 0.008), while there was no difference between the standard formula and MFGM and the breastfed group. There was a sex difference, with s-ferritin being higher in girls than in boys, a difference that was statistically significant for the breastfed and F19 groups. Error bars: 95% confidence intervals.

S-ferritin concentration was analyzed in a random sample consisting of 50 infants from each of the groups (Figure 3). There was no difference in s-ferritin results among the formula-fed groups, but values in the F19 group were significantly lower than in the breastfed group (*p* = 0.008). The SF and MFGM groups did not differ from the breastfed infants. There was a sex difference in s-ferritin in all groups, with girls having higher values, and values for girls differed significantly from boys for the breastfed and F19 groups. The number of infants with iron deficiency, defined as s-ferritin <12 μg/L, was two each in the breastfed and F19 groups and one each in the SF and MFGM groups.

## Discussion

In this randomized, double-blind, controlled multicenter study, we evaluated the safety and effects on infections and growth of two infant formulas, one supplemented with the probiotic bacterium *L. paracasei*, ssp*. paracasei* strain F19 and the other with the bovine MFGM fraction Lacprodan^®^ MFGM-10, as compared to the same unsupplemented standard infant formula. A breastfed group served as reference.

All three formulas were well accepted by the infants as well as the parents/care providers. Although the F19 group consumed slightly less formula per day than the SF group, all formula-fed groups had a higher average intake of formula during the intervention than recommended by the manufacturer, suggesting satisfactory adherence. This inference is further supported by the fact that of the 100 infants randomized in each group at 4 months, 92% of the F19 infants had detectable F19 in their stool, compared with none in the other two groups (data not shown).

Growth is an important safety outcome for any new ingredient used in infant formula (34). The formula-fed groups showed no difference from each other in weight, length, or head circumference z-scores at any time point. Compared with the breastfed reference group, at entry, infants assigned to the formula-fed groups were smaller, particularly with lower weight because of lower birth weight. This baseline difference is a reasonable explanation for why particularly early anthropometric measures (1–4 months) were higher for the breastfed group than for the formula-fed groups. A higher weight among breastfed infants compared to formula-fed infants early in life has been described in many studies (35, 36), as has also that this difference disappears after age 4–6 months. Average daily gain in weight, length, or head circumference did not differ among the formula-fed groups or between these infants overall and the breastfed group during the intervention. A previous report indicated that providing F19 during the weaning period does not affect body composition, growth, or any of the assessed metabolic markers at school age (37), and another study showed that supplementing an infant formula with the same MFGM fraction as used here did not affect growth (29). Overall, the anthropometric data for the MFGM and F19 groups did not differ from those of the SF group, taking the difference in size at birth into consideration. All three formula-fed groups showed growth patterns similar to formula-fed infants in other published studies and tracked with the World Health Organization growth charts.

We identified few AEs overall, with no differences within any of the AE categories among the formula-fed groups or between the breastfed and formula-fed groups during the intervention or the post-intervention period. The total number of AEs during the intervention was numerically higher among SF infants than in other groups, significantly so compared to the F19 group, but no groups differed after the intervention. Only 20 infants experienced SAEs, most commonly lower respiratory tract infections, with 16 episodes. Taking AEs and SAEs together, in no case could it be definitely concluded that the formula was the cause. A recent study from China from one of the study sites showed low iron status in both breastfed and formula-fed infants (38). In the present study, we found very few infants with iron deficiency, and overall iron status was satisfactory in all groups. The reason for this discrepancy is not known, but many factors affect iron status during early infancy including maternal iron status, cord clamping (39), and type of feeding (40).

Several randomized double-blind trials have assessed the effects on health of adding MFGM to infant formulas or diets for young children. In Belgian preschool children, a daily chocolate formula-milk supplemented with a phospholipid-rich MFGM concentrate resulted in a significantly reduced number of days with fever during the 4-month intervention period compared to the corresponding unsupplemented formula-milk (41). In a Peruvian double-blind randomized controlled trial healthy, primarily breastfed infants ages 6–11 months were given instant complementary food fortified with 1 RDA of multiple micronutrients, with either an MFGM-enriched protein fraction or skim milk powder (control group) as the protein source, daily for 6 months. The primary outcome was diarrhea. The groups showed no difference in the incidence of diarrhea, although the longitudinal prevalence of diarrhea was significantly lower in the MFGM compared with the control group. In a multivariate model adjusted for initial anemia and potable water facilities, the incidence of bloody diarrhea was lower in the MFGM group (30). In a Swedish study, term infants were randomized before the age of 2 months to formula with slightly reduced protein and energy content and supplemented with the same MFGM preparation or SF (28, 42, 43). A breastfed group served as reference. The formulas were used until age 6 months, and the infants were followed to age 12 months. During the intervention, the MFGM group had a lower incidence of acute otitis media than the SF group (1% vs. 9%, *p* = 0.034), lower incidence and longitudinal prevalence of antipyretic use, and a lower concentration of secretory IgG against pneumococci after vaccination (29), in agreement with previous findings of reduced infections.

In contrast, in a multicenter non-inferiority DBRCT on healthy term infants, Billeaud et al. evaluated the safety of two infant formulas, enriched with a lipid-rich or a protein-rich bovine MFGM fraction, respectively. At 14 days of age, the infants were randomized to receive standard infant formula (control), or one of the two experimental formulas until age 4 months. The primary outcome, weight gain, was non-inferior in the MFGM-lipid and MFGM-protein groups compared with the control group. Among secondary and exploratory outcomes, few between-group differences were observed. AEs and morbidity rates were similar across groups except for a higher rate of eczema with protein-rich MFGM compared to the other two groups (44). Of note, however, the total number of infants with eczema was low, and a Swedish study did not have a similar finding (45). A trial in India, evaluating the preventive effect against diarrhea of supplementation with a ganglioside concentrate during the second year of life, was inconclusive in the primary outcome of rotavirus diarrhea, and in secondary outcomes, including all-cause diarrhea (46). However, the study was underpowered because of a lower-than-expected diarrhea incidence.

In our study, compared to the breastfed group, the SF but not the MFGM and F19 infants had significantly more episodes of fever and days with fever during the intervention. Numerically the MFGM and F19 groups also had fewer episodes of fever and days with fever than the SF group, but not significantly. During the intervention, number of episodes of respiratory tract infections did not differ among the formula-fed groups or between these infants overall and the breastfed group; however, F19 infants had more episodes of upper and lower respiratory tract infections compared to the breastfed group. Furthermore, during the intervention, formula-fed infants did not differ among groups for skin affections, vomiting, or unscheduled visits/hospitalizations, although the F19 group used more antibiotics and had more unscheduled visits/hospitalizations than the breastfed group. After the intervention, only the F19 infants used more antibiotics than the breastfed group, while formula-fed groups showed no differences.

Of interest, during the intervention, the MFGM group did not have significantly more episodes of fever or number of days with fever, diarrhea, and use of antibiotics or unscheduled visits/hospitalization during the intervention than the breastfed group. This finding suggests health benefits particularly for this group. However, these positive effects were less obvious after the intervention when the MFGM group had more episodes of diarrhea than the breastfed infants. The incidence of diarrhea during the study period was lower than expected, making the study underpowered compared with the intention of the design. Furthermore, otitis media was not diagnosed because otoscopy check is not a clinical routine, nor was cognitive development assessed in this study. These results support the previous observation that supplementation with MFGM reduces the gap between formula-fed and breastfed infants with regard to infections. Preclinical studies have demonstrated that several proteins in the MFGM inhibit various pathogens, including *Escherichia coli*, rotavirus, and enterotoxins (25, 47–49). Further studies are needed to clarify the mechanisms behind the anti-infectious properties of MFGM.

Probiotics have been proposed to influence a wide range of health outcomes, presumably by altering the intestinal microbiota and, directly or indirectly, modulating the developing immune system (7). Studies on the probiotic F19 during the weaning period have shown a lower incidence of eczema at age 12 months (19) and increased capacity to raise immune responses to protein antigens (50). Another study, comparing infant formulas containing oligosaccharides with or without F19, showed a lower incidence of lower respiratory tract infections with synbiotics compared to prebiotics (20). In the present study, we found no significant difference in stool frequency, stool consistency (data not shown), or diarrhea episodes between the F19 and SF groups during the intervention. The two groups also did not differ for other primary or secondary outcomes. However, compared to the breastfed group, the F19 infants had more upper and lower respiratory tract episodes, as noted, along with use of antibiotics, and unscheduled visits/hospitalizations during the intervention period and more use of antibiotics after the intervention. Given the previous observation of lower frequency of lower respiratory tract infections in infants given F19 together with prebiotics (20) and less antibiotic use in infants fed F19 (50), the present observations are difficult to explain. However, besides these unexpected findings, overall, we observed no negative effects of adding probiotics (*Lactobacillus* F19) to infant formula, in agreement with previous studies. Of interest, consumption of probiotics during early infancy and increased infection risk among toddlers has been demonstrated (51), although the evidence is not conclusive.

Strengths of the present study are the large number of infants included and the double-blind randomized controlled design. Both the F19- and MFGM-enriched formulas met the primary safety endpoint with respect to anthropometrics compared to the SF group and also the breastfed reference group. In general, the formulas were well-tolerated with few AEs. The limitations of the study for investigating other outcomes include the number of sites, the absence of otitis media assessment, and the lack of cognitive development screening.

## Conclusions

Both the MFGM- and F19-supplemented formulas met the primary safety endpoint of weight gain that did not differ from infants assigned to the control formula. In general, the formulas were well-tolerated but showed no obvious positive effects on the health outcomes studied. Of note, however, during the intervention, the outcomes for the MFGM group were close to those of the breastfed group, supporting previous findings showing that supplementing infant formulas with MFGM narrows the gap between breastfed and formula-fed infants with respect to infections. Our findings provide support for further clinical evaluation of MFGM- or F19-enriched infant formulas.

## Data Availability

All datasets generated for this study are included in the manuscript/supplementary files.

## Author Contributions

XL, YP, BL, and OH: conceptualization. XL, YP, ZL, BL, and OH: principal investigators. HS: statistical analyses. XL, YP, BL, OH, and HS: methodology. XL, YP, OH, and BL: supervision. HS: visualization. XL, OH, HS, and BL: writing, original draft. XL, YP, ZL, BL, AH, HS, BC, and OH: writing, review and editing.

### Conflict of Interest Statement

The study was financially supported by Arla Foods amba, Arla Innovation Center, Skejby, Denmark and Arla Foods Ingredients, Viby, Denmark. Arla Foods amba provided the experimental and standard formulas. BC is an employee of Arla Foods amba and AH is an employee at Arla Foods Ingredients. XL, YP, ZL, HS, BL, and OH have received honoraria and/or travel grants or research grants from Arla Foods.

## Abbreviations

MFGM: milk fat globule membrane
F19: probiotic *L. paracasei* ssp. paracasei strain F19
SF: standard formula
AE: adverse event
SAE: serious adverse event.
